# Effect of Volume in Resistance Training on Inhibitory Control in Young Adults: A Randomized and Crossover Investigation

**DOI:** 10.3389/fpsyg.2018.02028

**Published:** 2018-10-29

**Authors:** Leonardo de Sousa Fortes, Manoel da Cunha Costa, Raphael José Perrier-Melo, Jorge Luís Brito-Gomes, José Roberto Andrade Nascimento-Júnior, Dalton Roberto Alves Araújo de Lima-Júnior, Edilson Serpeloni Cyrino

**Affiliations:** ^1^Graduate Program in Physical Education, Federal University of Pernambuco, Recife, Brazil; ^2^Graduate Program in Physical Education, University of Pernambuco, Recife, Brazil; ^3^Department of Physical Education, Federal University of Vale do São Francisco, Petrolina, Brazil; ^4^Graduate Program in Physical Education, State University of Londrina, Londrina, Brazil

**Keywords:** strength training, cognition, brain, attention, weight training

## Abstract

**Purpose:** The aim of the study is to compare the effect of resistance training volume on inhibitory control in young adults with previous experience in resistance training.

**Method:** All the 27 participants underwent 40-week experiment, divided in three training phases of 8-week duration. A washout period of 8 weeks between each of the training phases was carried out. The participants performed 1, 3, or 5 sets of the same exercises with equalized intensity (loading zones) and rest. Inhibitory control was assessed by the Stroop Test.

**Results:** Interaction effect was found for inhibitory control accuracy [*F*_(5,22)_ = 56.88, *p* < 0.01] and mean response time [*F*_(5,22)_ = 83.02, *p* < 0.01] for 3 sets (*p* = 0.01; ES = 0.6) and 5 sets (*p* = 0.01; ES = 0.8) when compared to 1 set.

**Conclusion:** In conclusion, 1 set of resistance training may provide insufficient volume stimulus for positive adaptation in inhibitory control when compared to 3 or 5 sets.

## Introduction

Cognitive function regards to an intellectual process that one becomes aware of perceiving or comprehend ideas (Verburgh et al., 2014). Attention, memory, inhibitory control, and cognitive flexibility are part of the cognitive function (Verburgh et al., 2014). In fact, aging is associated with reduced cognitive function (Zheng et al., 2016). On the other hand, as much as cognitive function is improved, information is processed faster and more accurate (Hanson et al., 2018). Interestingly, young adults with low levels of weekly physical activity present less cognitive function scores when compared to the active ones (sports or other physical exercise) (Verburgh et al., 2014). Thus, it is necessary to identify how to postpone or avoid the decrease on cognitive function in early ages.

Thereby, previous studies demonstrated that physical exercise might improve cognitive function (Weinberg et al., 2014; Northey et al., 2018). Nevertheless, predominance is noticed for studies that use aerobic exercise as intervention. Interestingly, effects of resistance training on inhibitory control remain slightly explored (Best et al., 2015; Iuliano et al., 2015). In fact, the purpose of those investigations was to analyze the effect of load on inhibitory control. In the study of Iuliano et al. (2015) elderly people underwent 12 weeks of resistance training with 50% of one repetition maximum (RM) intensity and no alterations on inhibitory control or selective attention were observed. In contrast, Jurakic et al. (2017) demonstrated that three sessions of resistance training (75% of 1RM) per week improved inhibitory control in elderly women. Opposing from those findings, Weinberg et al. (2014) found that memory was improved in a single session of resistance training for young adults (men and women), performed at 10 maximum repetition zone. However, it is important to emphasize that the disparity in results might be caused by different protocols adopted in each study.

Variables of training prescription such as intensity zone, resting, speed of execution, muscle time under tension, frequency, sets, and repetitions might be manipulated (American College of Sports Medicine[ACSM], 2009). The most important ones are intensity zone, resting, and speed of execution that comprise the component “intensity” in resistance training (Fonseca et al., 2014). Accordingly, frequency, sets, and repetitions comprise the volume (Schoenfeld et al., 2016). Resistance training volume has been associated with augmentation in the concentration of peripheral brain-derived neurotrophic factors (BDNFs) (Church et al., 2016) that positively influences cognitive function (Portugal et al., 2013). Thus, it seems that volume manipulation effect of resistance training on inhibitory control is relevant to be investigated.

Additionally, Best et al. (2015) found that in elderly women high volume of resistance training during 52 weeks produced an improvement in memory when compared to the ones who performed half of the volume prescribed. Conversely, Liu-Ambrose et al. (2010) found no difference on inhibitory control and attention in the two elderly women groups that carried out resistance training using two different volumes (once or twice-weekly) during 12 months. However, studies that analyzed the effect of resistance training volume on cognitive function components in elderly (Liu-Ambrose et al., 2010; Best et al., 2015) and young adults (Chang and Etnier, 2009) without cognitive deterioration did not equalize other prescription variables of resistance training, thereby, it is not possible to assure that volume affects inhibitory control. Moreover, studies that analyzed the effect of resistance training volume on inhibitory control were performed in elderly participants, hence, information about young population still lacks on the literature.

From a practical point of view, identifying alterations on inhibitory control from resistance training volume manipulation might ease the professional decisions in training centers. In regard that elderly people might improve inhibitory control due to high resistance training volume, the hypothesis of the present study is that similar results might occur in young adults. Thereby, the aim of the study is to compare the effect of resistance training volume on inhibitory control in young adults with previous experience in resistance training.

## Materials and Methods

### Experimental Design

**FIGURE 1:**
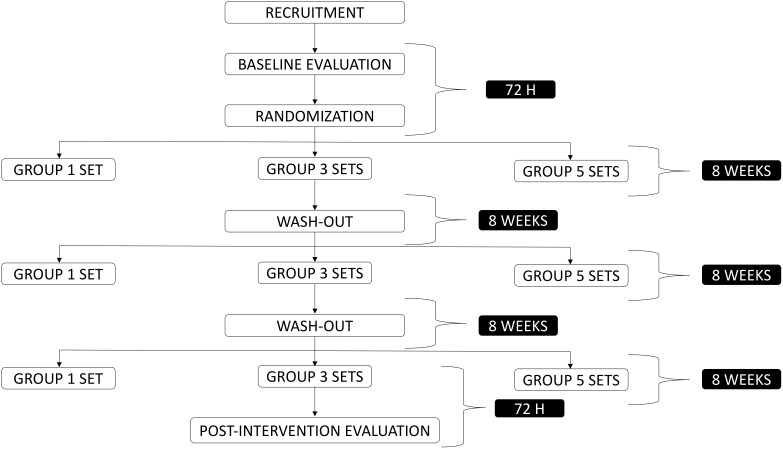
Experimental design of the investigation.

This is a crossover experimental study, controlled, randomized, and 40-week duration. The participants underwent 40-week intervention divided into three phases of resistance training that lasts 8 weeks (Group 1: participants underwent 1 set of resistance exercises; Group 2: participants underwent 3 sets of resistance exercises; Group 3: participants underwent 5 sets of resistance exercises). Also, the participants were submitted to an 8-week washout between each phase (Figure 1).

Inhibitory control, maximum muscular strength (1RM), 10 repetition maximum (10RM) and body composition were assessed 72–120 h before (pre-experiment) and 72–120 h following the last session (post-experiment) of each resistance training phase (1 vs. 3 vs. 5 sets). All the participants were oriented to not performing any kind of physical exercise 48 h before the assessments.

#### Experimental Conditions

**FIGURE 2:**
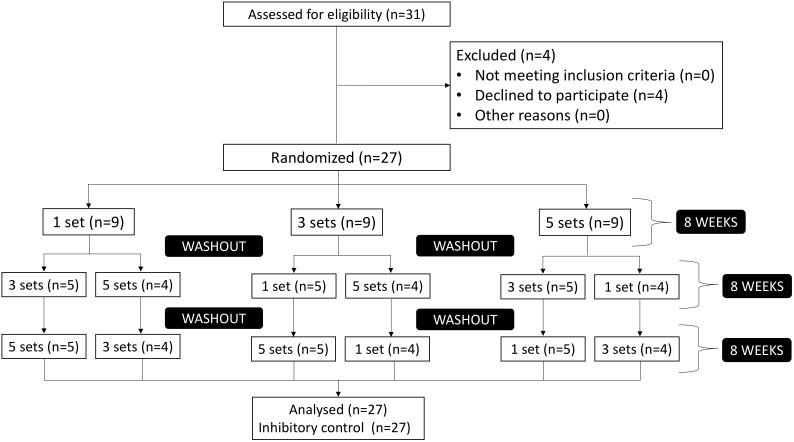
Flowchart of analyzed participants.

The participants performed 1, 3, and 5 sets of 8-week resistance exercises with equalized intensity (loading zones) and rest (Table 1). The three experimental conditions (1, 3, and 5 sets) were randomized and a washout period of 8 weeks was given between them (Figure 2). Thereby, the participants were allocated randomly in one of the three experimental conditions, following the 8-week period of training, another 8-week washout period was given before the next condition start off. This procedure was adopted repeatedly until all the three experimental conditions were performed for all the participants.

**Table 1:** Resistance training program.

Weeks	1 set	3 sets	5 sets
1–8	1 set of 10RM; 180 s between sets and exercises	3 sets of 10RM; 180 s between sets and exercises	5 sets of 10RM; 180 s between sets and exercises

*RM, repetition maximum.*

### Eligibility Criteria for Participants

All the participants had no injury background or were in use of any ergogenic substance for strength, muscular volume, or cognitive function in the last six months. The participants had to be practicing resistance training for at least 2 years. Only participants trained were recruited because they are adapted to the kind of physical exercise intervention (resistance training). The participants were oriented to not changing their routines, eating habits, and not engage in any other exercise program.

### Participants

The participants were recruited according the non-probability sampling, in total, 31 male volunteers aged from 18 to 30 participated in the experiment. The sample size provided statistical power >90%.

All the procedures were in agreement with Research Ethical in Humans Committee of Federal University of Pernambuco. The protocol was reviewed and approved by the Research Ethical in Humans Committee of Federal University of Pernambuco (CAAE – 47571415.9.0000.5208). All participants gave written informed consent in accordance with the Declaration of Helsinki.

#### Randomization

A simple randomization was carried out for the three experimental conditions (1, 3 or 5 sets). The random number table was generated on the www.randomizer.org site.

#### Sample Size

Sample size analysis was performed by G^∗^Power 3.1. It was adopted a power of 0.90, α = 0.05 and an effect size of 0.50 according to other studies (Fonseca et al., 2014; Ribeiro et al., 2016b). Thus, it was calculated that 24 participants were needed to carry out the study.

### Resistance Training Program (Intervention)

The training program was based on recommendations for resistance training in healthy adults (American College of Sports Medicine[ACSM], 2009) and composed by four exercises (bench press, leg press 45°, seated row, leg curl) performed three times per week (Monday, Wednesday, and Friday) during 8 weeks for each experimental approach (1 vs. 3 vs. 5 sets). All sessions were performed during the same period of the day (4 p.m.) to avoid circadian rhythm effect; also, the entirely session was supervised by experienced researchers in resistance training area.

Ten repetition maximum test was performed to define training intensity. The training was adjusted weekly (increase/decrease of 2–5 and 5–10% for upper and lower limbs, respectively) as recommended by Schoenfeld et al. (2016). In case of the participants performed more than 10 repetitions in two consecutive sessions, load (kg) was increased in the following week, whereas for the participants who were unable to perform at least 10 repetitions the load was decreased in the subsequent week.

Table 1 indicates the resistance training procedure in each condition (1 vs. 3 vs. 5 sets). The warm-up (1 × 20–25 reps with 50% of predicted 10RM and 1 × 10–15 reps with 80% of predicted 10RM, 3 min interval was adopted between sets) was performed for the first two exercises (bench press and leg press 45°) before each resistance training session (Fonseca et al., 2014). Also, during the washout period, all volunteers were instructed to maintain usual activities and abstain from physical exercise.

### Variables Measurements

#### Primary Outcome

##### Inhibitory control

Stroop Test (Parsons et al., 2011) was adopted for assessing inhibitory control, considered a component of cognitive function. Thereby, two assessments with an interval of 72 h were performed using the score means as baseline, as recommended by Bruce et al. (2016). Intraclass correlation coefficient and standard error of the measurement between the two baseline inhibitory control assessments were 0.97 and 2.6% for accuracy; 0.95 and 0.08 ms for response time, respectively. The tests were carried out on a full-hd screen (1800 × 1260 pixels) laptop (MacBook Pro, A1502 model, EUA). On the test, participants answered the word color or according its name, since the color of the words might be different from what is typed (e.g., the word “blue” might show up in “red” color, the word “green” in “blue” color, and so on). Stimuli of 62 words with 200 ms of interval were provided between response and new stimuli. Moreover, stimulus did not fade from the screen until any response. Stimuli vary between congruent (word and color have the same meaning), incongruent (word and color have different meaning), and control (colored rectangle with one of the colors of the test: red, green, blue, and black). For answering the questions, it was used the keys D (red), F (green), J (blue), and K (black). Colors were put on the respective keys: red color on the “D” key, green color on the “F” key, blue color on the “J” key, and black color on the “K” key. When the answer was correct, the stimulus disappeared and a new one was set. In case of incorrect answers, an “X” showed up on the screen and a new stimulus appeared subsequently. At the end of the test, the accuracy of the correct answers, mean response time, and errors were collected. All participants had total access to the result of their test. The evaluator was blind for all the assessments and had previous training for the test. The participants were familiar with the Stroop task. The resistance training volume may be able to increase the cortical activity in the frontal cortex, therefore, improving the inhibitory control performance.

#### Second Outcomes

##### Maximum muscular strength

Maximum muscular strength was determined by 1RM test. The exercises performed were bench press and leg press 45°. First, all the participants were familiarized with the 1RM test for reducing motor learning influences (Schoenfeld et al., 2016). Following, the participants were tested in similar conditions on the adopted protocol in two distinct sessions with intervals of 48 h. For each exercise, three attempts were made with intervals of 5 min between exercises and repetitions (Fonseca et al., 2014). The intraclass correlation coefficient and standard error of measurement between familiarization and 1RM tests were 0.99 and 4.1 kg for bench press and 0.99 and 6.8 kg for leg press 45°, respectively.

Additionally, warm-up (1 × 10–15 reps with 50% of predicted 1RM and 1 × 5–8 reps with 80% of predicted 1RM, adopting 3 min of interval between sets) was performed for each exercise (bench press and leg press 45°) before each muscular strength assessment; also, verbal encouragement was used throughout the test.

##### 10 repetition maximum

Intensity zone for 10RM was determined following the 10RM test. The exercises performed were bench press, leg press 45°, seated row, and leg curl.

All participants performed the 10RM test in two distinct sections with interval of 48 h. For each exercise, two attempts were made with intervals of 10 min between sets and exercises. Intraclass correlation coefficient and standard error of the measurement between familiarization and 10RM tests were 0.98 and 2.7 kg for bench press, 0.99 and 4.0 kg for leg press 45°, 0.97 and 3.6 kg for seated row, and 0.98 and 2.4 kg for leg curl, respectively.

Accordingly, warm-up (2 × 15–20 reps with 50% of predicted 1RM, adopting 120 s intervals between sets) for each exercise before performing 10RM test. Verbal encouragement was given throughout the 10RM test.

##### Body composition

Body mass (kg – portable scale PL 200, Filizola S.A., São Paulo, Brazil, accuracy of 0.1 kg) and height (professional stadiometer Sanny, São Paulo, Brazil, accuracy of 0.1 cm) were measured. Corporal density was measured using the technique of body scanning by the Dual X-ray Absorptiometry (DXA) (Hologic, Waltham, MA, United States). Participants were recommended to not performing any physical activity for at least 48 h and *ad libitum* hydration the day before. The participants remained in the supine position with arms besides the body and hands in neutral position. Feet and knees 10 cm away tied with a Velcro band for avoiding any movement that might interfere on the image visualization during the procedure. The analyzed variables were: free fat mass, fat mass, and body mass. DXA calibration followed the manufacturer recommendations as well as the measurements were performed for an experienced and blind for the experiment evaluator.

### Data Analysis

Shapiro–Wilk’s test was conducted to analyze data distribution. Levene’s test assessed the homoscedasticity of the groups. All the data are described as mean and standard deviation. Factorial repeated measures ANOVA 2 × 3 analyzed the interaction between time (pre vs. post) and intervention (1 set vs. 3 sets vs. 5 sets) for inhibitory control, maximum muscular strength, and body composition. Bonferroni’s *post hoc*, when necessary, identified statistical differences. Moreover, effect size (ES) revealed differences in a practical point of view. According to Rhea (2004), the following criteria were adopted: *d* < 0.35 = trivial, 0.35 ≤*d* > 0.8 = small effect size, 0.8 ≤*d* > 1.5 = moderate effect size, and *d* ≥ 1.5 = large effect size. All data were analyzed using the software SPSS 21.0, alpha level adopted was 5%.

## Results

From 31 participants, 27 completed the 40 weeks of the study as demonstrated in Figure 2.

### Accuracy and Response Time

The findings did not indicate differences for accuracy [*F*_(4,23)_ = 1.64, *p* = 0.34] and response time [*F*_(4,23)_ = 1.22, *p* = 0.41] among baseline and pre-measurements in all conditions (1, 3, and 5 sets).

**FIGURE 3:**
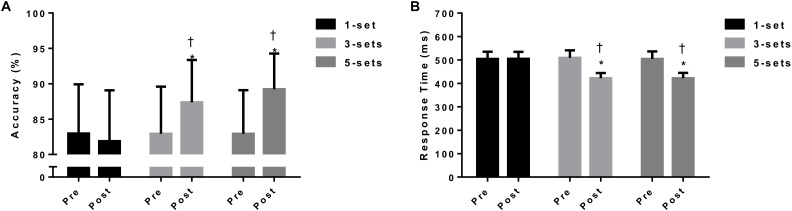
The panels **(A,B)** represent accuracy and response time findings according to interventions (1 vs. 3 vs. 5 sets). ^∗^*p* < 0.05 different from pre; ^†^*p* < 0.05 different from 1 set.

**FIGURE 4:**
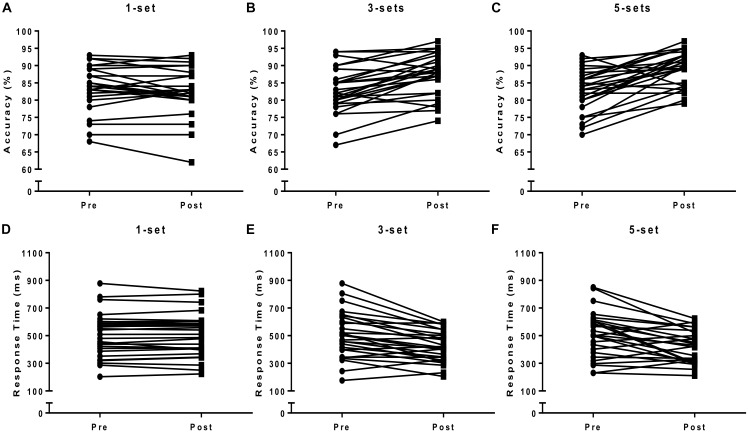
The panels **(A–F)** represent individual results to accuracy and response time.

Interaction effect (time vs. intervention) was found for accuracy [*F*_(5,22)_ = 53.00, *p* < 0.01]; 3 sets (*p* = 0.01; ES = 0.6) and 5 sets (*p* = 0.01; ES = 0.8) showed better performance when compared to 1 set (Figure 3A). Also, interaction (time vs. intervention) was observed for the mean response time [*F*_(5,22)_ = 86.10, *p* < 0.01]; 3 sets (*p* = 0.01; ES = 0.9) and 5 sets (*p* = 0.01; ES = 1.0) presented better scores when compared to 1 set (Figure 3B). The individual results pre- vs. post-experiment of accuracy and response time are presented in Figure 4. For the error rates, there was no main effect of group (1, 3, and 5 sets) or time (baseline, pre, and post-intervention) for commission errors [*F*_(5,22)_ = 1.49, *p* = 0.55] and omission errors [*F*_(5,22)_ = 1.23, *p* = 0.62] in the Stroop task.

### Maximum Muscular Strength

In regard of muscular strength, the findings indicated no differences in 1RM between baseline and pre-experimental measurements for bench press [*F*_(4,23)_ = 1.98, *p* = 0.27] and leg press 45° [*F*_(4,23)_ = 1.59, *p* = 0.35] for all conditions (1, 3, and 5 sets).

Results showed interaction (time vs. intervention) for bench press [*F*_(5,22)_ = 66.00, *p* < 0.01] (Table 2); 5 sets showed better performance when compared to 3 sets (*p* = 0.03; ES = 0.6) and 1 set (*p* = 0.01; ES = 1.6), as well as, 3 sets presented superior values when compared to 1 set (*p* = 0.01; ES = 1.1). Similar results for interaction (time vs. intervention) was found for leg press 45° [*F*_(5,22)_ = 63.1, *p* < 0.01] (Table 2), showing better results for 5 sets when compared to 3 sets (*p* = 0.02; ES = 0.7) and 1 set (*p* = 0.01; ES = 1.8); also, 3 sets was superior when compared to 1 set (*p* = 0.01; ES = 1.3).

**Table 2:** Mean and standard deviation of accuracy, response time, maximum muscular strength, and body composition (fat free mass, fat mass, and body mass) according to intervention (1 vs. 3 vs. 5 sets) and time (pre vs. post).

Variables	1 set (*n* = 27)	3 sets (*n* = 27)	5 sets (*n* = 27)	Effects	*F*	*p*
**Accuracy (%)**						
Pre	82.9 7.0	82.9 8.6	82.8 10.1			
Post	81.6 11.9	88.8 13.0^*,a^	90.2 10.5^*,a^	GxT	53.0	0.01
Δ%	-1.3 0.7	7.4 2.9	8.4 3.6			
**Response time (ms)**						
Pre	504.2 160.4	504.8 167.4	504.1 167.4			
Post	504.6 156.7	422.3 112.5^*,a^	422.1 116.8^*,a^	GxT	86.1	0.01
Δ%	-2.4 1.1	-17.2 7.7	-17.6 8.5			
**1RM BP (kg)**						
Pre	92.1 17.5	94.6 19.4	95.3 20.3			
Post	93.1 18.7	98.2 20.0^*,a^	101.3 17.4^*,a,b^	GxT	66.0	0.01
Δ%	1.8 1.3	5.5 2.4	9.1 4.6			
**1RM LP (kg)**						
Pre	206.2 31.3	202.8 34.2	208.0 36.6			
Post	205.9 37.8	210.0 35.1^*,a^	218.5 28.4^*,a,b^	GxT	63.1	0.01
Δ%	-1.9 1.1	4.3 1.7	7.5 2.6			
**Fat free mass (kg)**						
Pre	61.5 7.2	62.2 8.4	63.7 8.9			
Post	61.4 8.2	64.7 9.6^*,a^	65.1 7.3^*,a^	GxT	40.2	0.02
Δ%	-1.7 0.7	4.5 2.6	5.1 2.5			
**Fat mass (kg)**						
Pre	15.2 7.0	15.7 6.8	16.0 7.7			
Post	15.8 8.1	13.6 7.9^*,a^	12.1 6.7^*,a^	GxT	51.3	0.01
Δ%	0.9 0.5	-6.7 3.3	-8.2 4.1			
**Body mass (kg)**						
Pre	77.6 9.1	78.2 9.8	79.3 9.6			
Post	77.0 11.8	78.6 9.8	77.1 10.4	GxT	3.6	0.44
Δ%	-1.7 0.8	1.0 0.7	-1.3 0.7			

*GxT, group vs. time interaction; BP, bench press; LP, leg press; Δ%, percentage change; ^∗^p < 0.05 vs. pre-intervention; ^a^p < 0.05 in relation to “1 set”; ^b^p < 0.05 in relation to “3 sets”.*

### Body Composition

In regard to body composition, findings did not indicate differences for fat free mass [*F*_(4,23)_ = 2.07, *p* = 0.21], fat mass [*F*_(4,23)_ = 1.16, *p* = 0.48], and body mass [*F*_(4,23)_ = 1.79, *p* = 0.42] between baseline and pre-experimental measurements for all conditions (1, 3, and 5 sets).

In regard to fat free mass, results revealed interaction between time vs. intervention [*F*_(5,22)_ = 40.22, *p* < 0.02] (Table 2); 5 sets presented better conditions when compared to 1 set (*p* = 0.01; ES = 0.8). According fat mass, interaction (time vs. intervention) was observed [*F*_(5,22)_ = 51.31, *p* < 0.01] (Table 2), presenting reduction in the 5 (*p* = 0.01; ES = 1.2) and 3 sets (*p* = 0.03; ES = 0.6) condition when compared to 1 set. However, in respect to body mass, the results did not indicate interaction [*F*_(5,22)_ = 3.63, *p* = 0.44] (Table 2).

## Discussion

To the best of our knowledge, the present study is the first that analyzed the effect of training volume on inhibitory control in trained young adults. Our hypothesis consisted that greater volume would cause superior results in higher volumes when compared to the lower ones. Therefore, according to the results, our hypothesis was confirmed due to positive effect of higher volumes of resistance training on inhibitory control.

The results of this study revealed better scores for the participants that performed 3 and 5 sets of the experimental conditions when comparing with 1 set, corroborating with another previous study (Best et al., 2015), even though it was conducted with elderly women without experience in resistance training. Moreover, resistance training volume seems to positive affect inhibitory control. Thus, this phenomenon is explained by increment of BDNFs. Systematic reviews indicate that increased muscular contractions, independently of load, is correlated with augmented concentrations of BNDFs (Huang et al., 2014; Dinoff et al., 2016). Moreover, BDNFs have been associated with tryptophan reduction in the brain, responsible for the neurotransmitter serotonin (Portugal et al., 2013). Thus, once serotonin levels are increased in the brain the greater is the lethargic state, which might cause inhibitory control attenuation. Thereby, increased resistance training volume seems to inhibit, even indirectly, concentrations of cerebral serotonin. In addition, another possible explanation for improved accuracy on the Stroop Test is the inflammatory markers reduction and augmented anti-inflammatory cytokines. Chupel et al. (2017) revealed reduction in the C-reactive protein levels and tumor necrosis alpha (TNF-alpha) with concomitant augmentation of interleukin-10 (IL-10) and improvement of cognitive function in older women that underwent a resistance training program. Also, recent findings showed resistance training might decrease TNF-alpha and C-reactive protein concentration (Ribeiro et al., 2016b; Fedewa et al., 2017). Therefore, these inflammatory markers seem to be associated to increased cerebral ammonia concentration that is inversely proportional with cognitive function performance (Deslandes et al., 2009).

The results of this present study indicated an improvement in the response time mean of inhibitory control in experimental conditions of 3 and 5 sets when compared to 1. Thereby, it is reasonable to assume that resistance training volume positively affects the performance of inhibitory control (Brush et al., 2016). It seems that augmentation on long-term muscular contractions might cause cerebral neurogenesis (Portugal et al., 2013), defined as formation of new neurons (Deslandes et al., 2009). Thus, once brain neurons are increased, speed of information processing might be optimized (Portugal et al., 2013), which could explain the improvement on the response time of the Stroop test for 3 and 5 sets condition.

According to maximum muscular strength, results revealed dose-response effect for the training volume. Five sets condition produced greater increase in maximum muscular strength when compared to 3 and 1 set. Likewise, 3 sets produced an increase in maximum muscular strength when compared to 1 set. Those results corroborate with a systematic review with meta-analysis conducted for Grgic et al. (2018) that demonstrated high resistance training volume is associated with increased muscular strength.

Regarding body composition, the results of the present study indicates dose-response effect for training volume on fat free mass and fat mass; however, the same did not occur for body mass. Moreover, our findings corroborate with other studies (Ribeiro et al., 2016a,b; Schoenfeld et al., 2016). In fact, increased resistance training volume is rather associated with augmentation of fat free mass and decrease in fat mass (Schoenfeld et al., 2016). Considering an increment in fat free mass and fat mass attenuation, it is common not finding any alteration in body mass following a resistance training program as our results indicate.

Although the present study revealed interesting results that might add information on the scientific literature, it presents some limitations that should be mentioned. Magnetic resonance was not utilized for getting brain images that could explain the improvement of inhibitory control in experimental conditions (3 and 5 sets). Also, it was not possible to use electroencephalogram for analyzing the behavior of brainwaves (alpha and theta) during rest. The BDNFs and inflammatory markers were not analyzed, thus, our finding should be treated with caution. Moreover, we highlight the absence of a control condition (eight-week without resistance training) as a limitation. However, the comparisons between baseline and pre-experiment measures of every experimental condition (1, 3, and 5 sets) indicate that eight weeks without resistance training did not affect inhibitory control, 1RM, and body composition, showing that eight-week washout period (without resistance training) was enough to maintain the participants in the same conditions as the baseline. Nevertheless, our research used the crossover design with repeated measures and washout periods that might reduce study limitations. A strong point that should be mentioned are the two baseline measurements of the Stroop Test that according to Bruce et al. (2016) if no differences are found, it might reduce random error of neurocognitive measurements.

## Conclusion

1 set of resistance training may provide insufficient volume stimulus for positive adaptation in inhibitory control when compared to 3 or 5 sets. Nonetheless, experimental conditions 3 and 5 sets demonstrated similar findings. Thereby, in a practical point of view, if the aim is to generate positive adaptation in the inhibitory control in young adults, 3 or 5 sets of resistance training program during 8 weeks could be enough.

## Author Contributions

LdSF conducted the experiment and wrote the paper. MC, RP-M, JB-G, and JN-R reviewed the paper. DdL-J conducted data analysis and revised the paper. EC guided the project.

## Conflict of Interest Statement

The authors declare that the research was conducted in the absence of any commercial or financial relationships that could be construed as a potential conflict of interest.
